# Immunogenicity and Safety of Inactivated Sabin-Strain Polio Vaccine “PoliovacSin”: Clinical Trials Phase I and II

**DOI:** 10.3390/vaccines9060565

**Published:** 2021-05-29

**Authors:** Anastasia Piniaeva, Georgy Ignatyev, Liubov Kozlovskaya, Yury Ivin, Anastasia Kovpak, Alexander Ivanov, Anna Shishova, Liliia Antonova, Yusuf Khapchaev, Irina Feldblium, Olga Ivanova, Aleksandra Siniugina, Aydar Ishmukhametov

**Affiliations:** 1 Chumakov Federal Scientific Center for Research and Development of Immune-and-Biological Products RAS (FSBSI “Chumakov FSC R&D IBP RAS”), 108819 Moscow, Russia; ignatjev_gm@chumakovs.su (G.I.); lubov_i_k@mail.ru (L.K.); ivin_uu@chumakovs.su (Y.I.); kovpak_aa@chumakovs.su (A.K.); ivanov_ap@chumakovs.su (A.I.); shishova_aa@chumakovs.su (A.S.); antonova_lp@chumakovs.su (L.A.); hapchaev_uh@chumakovs.su (Y.K.); ivanova_oe@chumakovs.su (O.I.); sinyugina@chumakovs.su (A.S.); ishmukhametov@chumakovs.su (A.I.); 2 Institute for Translational Medicine and Biotechnology, First Moscow State Medical University (Sechenov University), 117418 Moscow, Russia; 3 Perm State Medical University Named after Academician E.A. Wagner, 614990 Perm, Russia; irinablum@mail.ru

**Keywords:** Sabin strain, inactivated vaccine, IPV, poliovirus, clinical trial

## Abstract

Global polio eradication requires both safe and effective vaccines, and safe production processes. Sabin oral poliomyelitis vaccine (OPV) strains can evolve to virulent viruses and result in poliomyelitis outbreaks, and conventional inactivated poliomyelitis vaccine (Salk-IPV) production includes accumulation of large stocks of neurovirulent wild polioviruses. Therefore, IPV based on attenuated OPV strains seems a viable option. To increase the global supply of affordable inactivated vaccine in the still not-polio free world we developed an IPV made from the Sabin strains–PoliovacSin. Clinical trials included participants 18–60 years of age. A phase I single-center, randomized, double-blind placebo-controlled clinical trial included 60 participants, who received one dose of PoliovacSin or Placebo. A phase II multicenter, randomized, double-blind, comparative clinical trial included 200 participants, who received one dose of PoliovacSin or Imovax Polio. All vaccinations were well tolerated, and PoliovacSin had a comparable safety profile to the Placebo or the reference Imovax Polio preparations. A significant increase in neutralizing antibody levels to polioviruses types 1–3 (Sabin and wild) was observed in PoliovacSin and Imovax Polio vaccinated groups. Therefore, clinical trials confirmed good tolerability, low reactogenicity, and high safety profile of the PoliovacSin and its pronounced immunogenic properties. The preparation was approved for clinical trials involving infants.

## 1. Introduction

The history of effective poliomyelitis control goes back more than 60 years since the development of vaccines from inactivated wild and live attenuated strains, i.e., inactivated polio vaccine (IPV) and oral polio vaccine (OPV). The use of the vaccines allowed not only to reduce the incidence of poliomyelitis worldwide but also to start implementing a program to eradicate the disease. At the 41st World Health Assembly in 1988, a resolution (WHO 41.28) was adopted, which committed WHO member states to the global eradication of polio and approved the eradication strategy, which included routine and supplementary immunization practices and epidemiological surveillance [1]. OPV became the vaccine of choice for the Global Polio Eradication Initiative (GPEI).

However, the live OPV turned out to be potentially dangerous. It can cause vaccine-associated paralytic poliomyelitis (VAPP) in approximately one case per 2.7 million doses of OPV. Rare VAPP cases are registered in OPV recipients or their unvaccinated contacts [2]. Another problem is the ability of Sabin strains to evolve in the organism of the recipient or contact persons that leads to the formation of vaccine-derived polioviruses (VDPV) with restored neurovirulent properties [3].

Nevertheless, during the last 30 years, the GPEI achieved incredible success: the incidence of poliomyelitis caused by wild polioviruses (PV) was thousandfold reduced [4], the eradication of wild polioviruses of types 2 [5] and 3 [6] was recognized, five of the six WHO regions were certified as “polio-free” [7,8,9,10,11].

Unfortunately, during the last five years situation with poliomyelitis has changed: wild polioviruses type 1 continue to circulate in Afghanistan and Pakistan [12]; circulating VDPVs type 2 caused large polio outbreaks through the African continent and recently in Tajikistan; and VDPVs types 3 were detected in China and Somalia in recent years [13]. This poses a threat to polio-free countries. Russia, which has a tight migration bonds with Central Asian countries, can easily become a target of virulent poliovirus importation, as has already occurred in 2010 [14]. All these facts increase the importance of commitment for polio vaccination using trivalent vaccines.

As a part of preparation for the global certification of a polio-free world, the GPEI decided to exclude the Sabin 2 as the cause of the most VDPVs from immunization. It was recommended to switch from the use of trivalent OPV (from PV types 1, 2 and 3) to a bivalent (from PV types 1 and 3) by May 2016. The use of a bivalent vaccine should be preceded by the administration of at least one dose of IPV for routine immunization worldwide to maintain immunity against PV type 2 [15].

Further steps of preparation for the polio-free world include a transition from OPV to IPV and global containment of polioviruses [16].

The technology of IPV production has undergone many changes since its creation in 1953 by Jonas Salk [17]. The current process of producing Salk-IPV includes several stages, from the process of cultivating sensitive cell lines, such as Vero, and harvesting the virus, to chromatographic purification and inactivation with formaldehyde [18]. The production process of conventional Salk-IPV involves accumulation of huge stocks of high titered neurovirulent wild PV strains, which poses a threat to biosecurity. The use of Sabin PV strains for IPV production (Sabin-IPV) would significantly reduce this risk.

There were several procedures developed for Sabin-IPV production [19,20,21,22,23]. Moreover, the use of Sabin-IPV for routine immunization in Japan provided an example of successful implementation [23].

Here we present the development and clinical trials of PoliovacSin, a purified concentrated inactivated liquid vaccine against poliomyelitis from attenuated Sabin strains. The data obtained in the phase I trial showed the good tolerability, low reactogenicity and high safety profile of the PoliovacSin compared to Placebo. The data obtained in the phase II trial showed the good tolerability, high safety profile and pronounced immunogenic properties of the PoliovacSin in comparison with the Imovax Polio as a reference preparation.

## 2. Materials and Methods

### 2.1. Cells and Viruses

Vero cell line obtained from WHO (10–87) was used. Vero cells for PV accumulation during the production process were maintained in EMEM (FSBSI “Chumakov FSC R&D IBP RAS”, Moscow, Russia), supplemented with fetal bovine serum (FBS, 5%).

Hep2c cell line originated from NIBSC (UK, South Mimms) was used for neutralization test. Cells were maintained in EMEM with doubled amino acids and vitamins (FSBSI “Chumakov FSC R&D IBP RAS”, Moscow, Russia), supplemented with 5% FBS, streptomycin (0.1 mg/mL), and penicillin (100 units/mL) (PanEco, Moscow, Russia).

Sabin PV type 1 (LSc 2ab), Sabin PV type 2 (P712 Ch 2ab) and Sabin PV type 3 (Leon 12a_1_b) were used for vaccine production and neutralization test. Wild PV type 1 (strain Mahoney), type 2 (strain MEF), and type 3 (strain Saukett) were used for a neutralization test as well. All strains originated from WHO or NIBSC and were received for OPV production or laboratory testing.

### 2.2. Biosafety and Biosecurity Measures

FSBSI “Chumakov FSC R&D IBP RAS”, Moscow, Russia, is an OPV producer and scientific research institute. It is fully accredited by the national authorities for work with BSL 1-3 agents. And now has a Certificate of participation in poliovirus containment (RUS-CP-20191202-007) issued by GCC.

### 2.3. Vaccine

#### 2.3.1. Upstream Processes

Vero cells for the vaccine production were expanded in disposable bioreactors (Cytiva, Marlborough, MA, USA, and Sartorius AG, Gottingen, Germany) on microcarriers Cytodex 1 (Cytiva, Marlborough, MA, USA) at 37 °C with pH of 7.2 and dissolved oxygen of 50%. After reaching the confluence of Vero cells, the medium was exchanged to medium 199 (FSBSI “Chumakov FSC R&D IBP RAS”, Moscow, Russia) after washing with Hanks buffer solution. The cultivation of the virus was carried out at 34 °C until complete degeneration of the Vero cell monolayer.

#### 2.3.2. Downstream Processes

PV-containing medium was collected, clarified using filter cascade, and concentrated by tangential flow filtration. PVs were purified from cellular and serum proteins and nucleic acids by two-step chromatography: size exclusion (SEC) and ion exchange (IEX).

After IEX virus was inactivated during 13-days incubation with formaldehyde (final concentration 0.025%, *m/v*) at 37 °C. D-antigen content was estimated using ELISA [24] in three monovalent bulks (Sabin types 1-2-3), then a combined trivalent final bulk was formulated in a ratio 15-15-50 DU/dose followed by sterilizing filtration. Vaccine was aliquoted in ampoules of 0.5 mL per single human dose.

#### 2.3.3. PoliovacSin Composition

Each 0.5 mL dose is formulated to contain: (1) Active Ingredients: inactivated polioviruses, attenuated inactivated Sabin strains of types 1, 2, 3, with D antigen content: type 1 at least 15 DU; type 2 at least 15 DU; type 3 at least 50 DU. (2) Excipients: polysorbate 80, residual formaldehyde, medium 199 (containing in particular amino acids, mineral salts, vitamins, glucose and water for injections), buffer solution (sodium hydrophosphate, sodium dihydrophosphate, sodium chloride, water for injection).

It does not contain antibiotics and an adjuvant. PoliovacSin was developed as a vaccine for use in infants from 3 months of age with minimal risk of side effects.

### 2.4. Study Design and Participants

The single-center, randomized, double-blind, placebo-controlled phase I of clinical trials was conducted at the Healthcare Unit No. 163 of FMBA of Russia (Novosibirsk, Russia). The duration of the study, including laboratory tests, was from January to March 2018. The purpose of this study was to evaluate the safety of the PoliovacSin (lot IPV-04-09.17) in comparison with the placebo (physiological saline) preparation in participants 18–60 years of age. At the screening stage, 99 participants aged 18–60 years signed informed consent. Of these, 60 participants were included in the study based on clinical, laboratory and instrumental examinations. They were randomly assigned 1:1 to receive the PoliovacSin or Placebo once in a dose of 0.5 mL.

The phase II of clinical trials was a multicenter, randomized, double-blind, comparative clinical trial. The study was conducted in two clinical centers: the Healthcare Unit No. 163 of FMBA of Russia (Novosibirsk, Russia) and Perm State Medical University named after Academician E.A. Wagner (Perm, Russia). The duration of the study, including laboratory tests, was from June to August 2018. The purpose of this study was to evaluate the safety and the immunogenicity of the PoliovacSin (lot IPV-04-09.17) in comparison with the Imovax Polio (reference preparation) (SANOFI PASTEUR, S.A., lot P3B636M) in participants of 18–60 years of age. In total, 200 healthy participants were enrolled and randomly assigned to receive PoliovacSin or Imovax Polio once in a dose of 0.5 mL.

The study protocols were conducted in accordance with Declaration of Helsinki guidelines, ICH GCP and Russian regulations. The study protocol, the informed consent form, and other documents requiring preliminary consideration were approved by the Ethics Committee under the Ministry of Health of the Russian Federation (No. 162 dated 23 January 2018 and No. 168 dated 24 April 2018). PoliovacSin was produced by the FSBSI “Chumakov FSC R&D IBP RAS”, which was the sponsor of the clinical trials.

### 2.5. Randomization

Randomization was performed using the sealed envelope system. The distribution of participants into groups was carried out using a random number generator (Microsoft Office Excel, the RAND function, followed by sorting in ascending order). The participant included in the study was assigned a randomization two-digit number incrementally (in order) b.

After assigning a randomization number to participants, a sealed envelope with a participant number was given to a healthcare worker who administered the vaccine. The envelope contained a tear-off sticker with the participant randomization number and the name of the corresponding preparation (placebo, PoliovacSin or Imovax Polio). The participants did not know which vaccine they were receiving. After the vaccination, a separate physician observed the participant for possible adverse events. The laboratory specialist who tested the sera was provided only with the code number of the participant and thus was also blinded.

### 2.6. Safety Assessment

After the vaccination, the following parameters were assessed: neurological status, biochemical blood parameters, general urine parameters, IgE levels, body temperature, and any reactions or adverse events.

The severity of adverse events was scored on a 4-point scale in accordance with the standards required by the Russian regulations.

Grade 0 (no reaction): local reactions—no symptoms, systemic reactions—no symptoms, temperature—up to 37.0 °C;

Grade 1 (mild adverse event): an adverse event, easily tolerated by the participant, causing minimal inconvenience and not interfering with daily activities; local reactions—hyperemia up to 50 mm in diameter or infiltrate up to 25 mm, systemic reactions—mild symptoms, temperature–from 37.1 °C to 37.5 °C;

Grade 2 (moderate adverse event): an adverse event that causes discomfort and interferes with daily activities; local reactions—hyperemia of more than 50 mm or an infiltrate of 26–50 mm, systemic reactions—symptoms that significantly impair normal daily activity, temperature—from 37.6 °C to 38.5 °C;

Grade 3 (severe adverse event): an adverse event that is incompatible with normal daily activities.

In all cases of appearance of any side effects, the participants had to inform the research physician by phone or at the next visit to the clinic; local reactions–infiltrate more than 50 mm, systemic reactions–symptoms that impede normal daily activity, temperature–more than 38.6 °C;

On the day of vaccination, the research physician performed a clinical examination of all participants before vaccination. Immediately before vaccination, the thermometry was performed (10 min before injection), as well as 2 h after vaccination. In phase I of clinical trials, the participants were observed in the hospital for 24 h on the day of vaccination. In phase II of clinical trials after the physical examination, the participants could leave the clinical center.

For the first 7 days, participants visited the clinical center every day. Repeated visits were made on the 14th and 28th day after the vaccination. For days 8–13 and 15–27 the participants were required to record the injection site (local) adverse events (e.g., pain, redness, or swelling) and systemic adverse events (e.g., fever, vomiting, headache, etc.) on the diary card.

### 2.7. Blood Sampling

Blood samples for immunogenicity assessment were collected on days 0 and 28 after the vaccination. Additionally, on days 0, 3, 14 and 28, the vaccination blood samples were collected for clinical and biochemical tests, as well as for IgE assay.

### 2.8. Neutralization Test (NT)

Poliovirus neutralizing antibodies (nAB) were determined in sera via microneutralization test in Hep2c cells against PV vaccine strains Sabin types 1–3 and wild strains Mahoney, MEF and Saukett using standard protocol [25].

### 2.9. Statistical Analysis

Descriptive statistics were used for all data: the data were checked for normality using the Shapiro–Wilk W test. In the case of a normal distribution, the mean and standard deviation were calculated. In cases of data inconsistency with normality, the median and quartile range were calculated. One-way analysis of variance (ANOVA) or nonparametric Mann–Whitney test was used for data comparison. Differences were determined at a significance level of *p* < 0.05. Statistical analysis was performed using OriginPro 8 (OriginLab Corp., Northampton, MA, USA).

## 3. Results

### 3.1. Safety Assessments—Phase I

In the phase I trial a total of 99 participants, 18–60 years old, were screened, and 60 were recruited into the study; 36 participants then withdrew from study and 3 were not eligible due to inclusion criteria.

**Figure 1:**
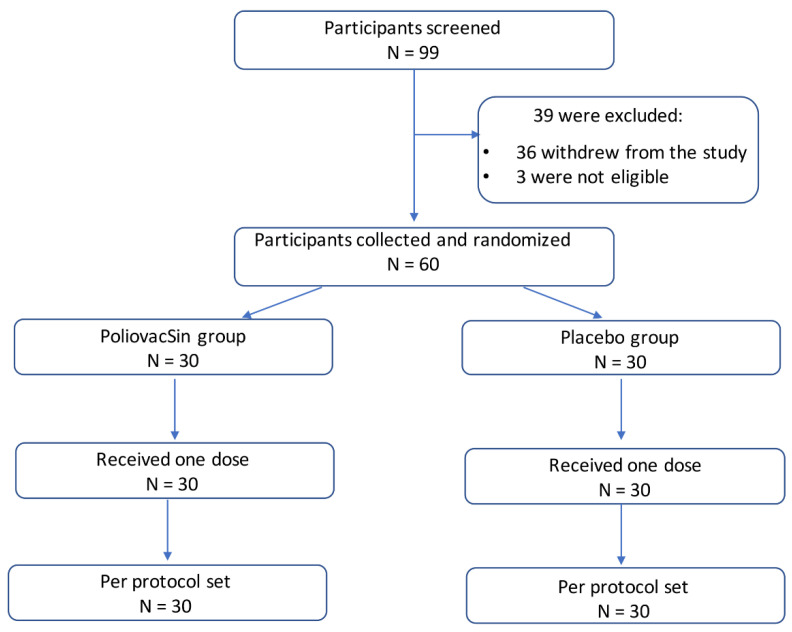
Participant flow through the study. Phase I.

Among all participants, 30 in the PoliovacSin group and 30 in the Placebo group received one dose of vaccine or placebo (Figure 1). All subjects were Caucasian. The groups did not differ significantly in age, sex, height, or weight (Table 1).

After the vaccination, a total number of participants developing adverse events (local or systemic) was 3 (5.0%): 2 (3.33%) in the PoliovacSin group and 1 (1.67%) in the Placebo group (Table 2). The reactions were mild (Grade 1), and lasted, in general, no more than 2 days. Reported systemic adverse event (Grade 2) was not related to vaccination.

### 3.2. Safety Assessments—Phase II

In the phase II trial a total of 213 participants, 18–60 years old, were screened, and 200 were recruited into the study; 13 were not eligible due to inclusion criteria.

**Figure 2:**
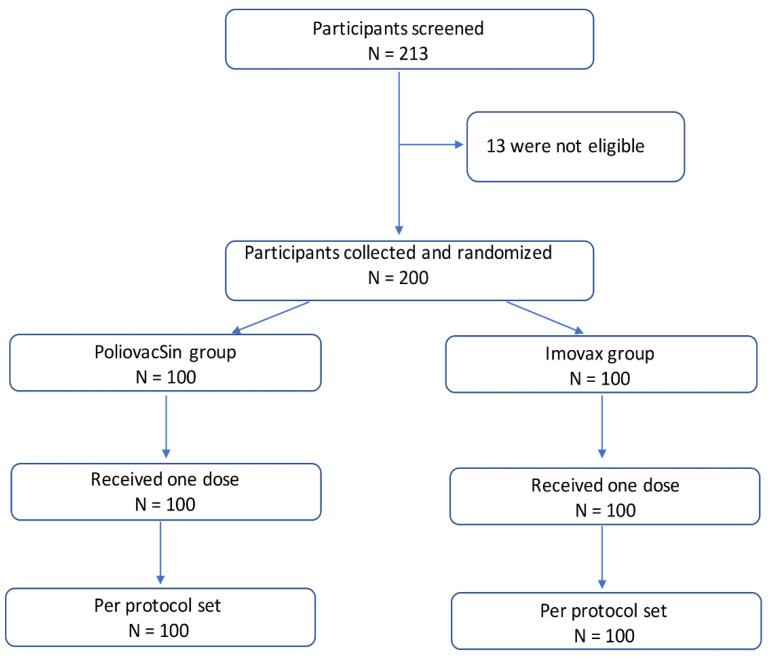
Participant flow through the study. Phase II.

Among all participants, 100 in the PoliovacSin group and 100 in the Imovax group received one dose of PoliovacSin or Imovax Polio vaccine (Figure 2). All subjects were Caucasian. The groups did not differ significantly in age, sex, height, or weight (Table 3).

In total, 26 local or systemic reactions were registered during the first 7 days after vaccination, of which 13 local (6.5%) reactions, and 13 systemic (6.5%) reactions (Table 4) Most of the reactions were mild (Grade 1). Two grade 2 reactions, recorded in PoliovacSin group included myalgia and pain at the injection site, resolved quickly. All reported systemic adverse events were not related to vaccination. None of the identified local or systemic reactions required prescription drug therapy.

Most of the participants had no local or systemic reactions during the period from 8 to 27 days after vaccination. In total, 3 systemic (3.0%) reactions were detected in two participants vaccinated with PoliovacSin. On the 22nd day after vaccination, one of the participants developed a mild viral respiratory infection; and on the 7th day, one of the participants had a concussion sustained in domestic conditions. Both reactions were considered unrelated to the previous vaccination.

Moreover, vaccination with either PoliovacSin to Imovax Polio did not have a negative effect on the main clinical and biochemical blood parameters, IgE levels, and general urine characteristics.

### 3.3. Immunogenicity Assessment—Phase II

**Figure 3:**
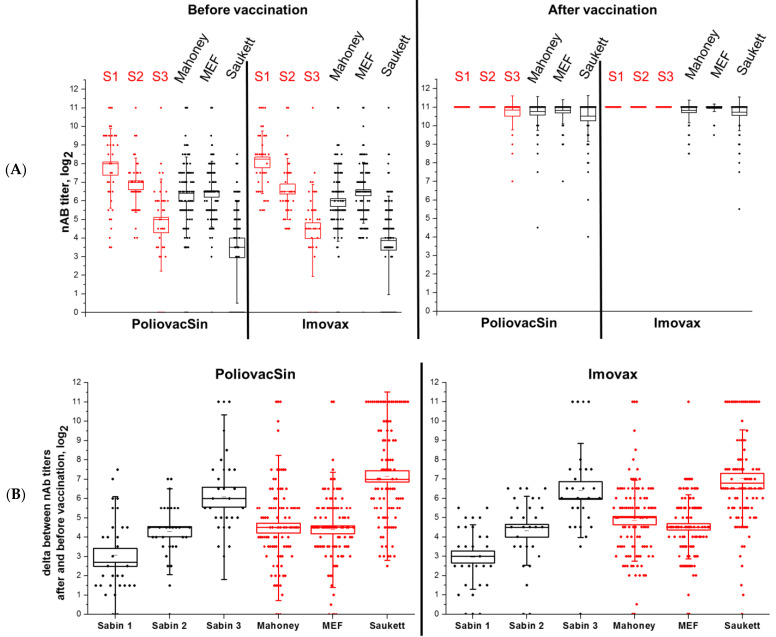
nAB titers against vaccine PV strains Sabin 1-3 and wild PV strains Mahoney (type 1), MEF (type 2) and Saukett (type 3) detected in the serum of Phase II participants before and after vaccination: (**A**) absolute values (**B**) Delta between the nAB titers against before and after vaccination. The differences between delta values of nAB titer induced by PoliovacSin and Imovax polio are statistically insignificant (Mann–Whitney test, *p* < 0.05).

Blood serum collected from all 200 participants before and after vaccination was tested in NT in Hep2c cells against vaccine poliovirus strains Sabin 1–3 (N = 73) and wild poliovirus strains Mahoney, MEF, and Saukett (N = 200). The nAB titers are presented in Figure 3.

All participants except one had nAB against at least one poliovirus type before the 1st vaccination. Moreover, 66 participants did not have detectable titers against wild PV type 3 (strain Saukett). However, the PoliovacSin and Imovax Polio groups did not differ statistically by the nAB titers against any virus used (Mann–Whitney, *p* > 0.05) before vaccination. The fact indicated that all the participants had been vaccinated earlier in their lives. As Salk-IPV is being used in Russia for routine immunization only since 2008, all the participants most likely have been vaccinated with trivalent OPV.

After the vaccination all participants had nAb against all tested viruses, vaccine and wild, in very high titers: 68 in PoliovacSin group and 80 in Imovax Polio group showed titers ≥ 1:1024 against all viruses. The titers against strains Sabin 1, Sabin 2 and Mahoney did not differ significantly between the two groups. On the other hand, the titers against strains Sabin 3, MEF, and Saukett tend to be a little lower in the group vaccinated with PoliovacSin (Mann–Whitney, *p* < 0.05). However, the differences between the nAB titers induced by PoliovacSin and Imovax Polio before and after vaccination did not differ significantly (Mann–Whitney, *p* < 0.05).

## 4. Discussion

Starting from 2016, developed and developing countries, especially those using only OPV for routine immunization, had to switch their routine immunization to IPV-OPV or to full IPV schedules following the WHO recommendations [26]. This led to an acute global IPV supply shortage. At the same time, strict precautions taken to avoid the possibility of live polioviruses escape from the production sites provided additional stress on the vaccine production from wild polioviruses and development of the novel technologies. In order to increase the global supply of affordable IPV vaccine, several manufacturers have focused on Sabin-IPV development in recent decades.

The development of IPV production technology based on attenuated Sabin strains began in the 1980s [27]. Many developers have already reported successful results of phase 1, 2, and 3 clinical trials, and some manufacturers have published the results of phase 4 clinical trials. Nevertheless, by 2021 only two countries China and Japan use Sabin-IPV in the expanded program on immunization (EPI) [28]. Sabin-IPV was introduced for infant immunization in Japan in 2012 (the first licensed Sabin-IPV in the world) [29]; and Sabin-IPV produced by the Institute of Medical Biology, Chinese Academy of Medical Sciences (IMBCAMS) has been introduced into the Chinese EPI schedule in 2016 [28].

Moreover, a long-term population study conducted in Japan showed that Sabin-IPV induced a sufficient level of neutralizing antibody [30] and protective antibodies against currently circulating and reference wild poliovirus strains and most vaccine-derived poliovirus strains [31].

As with many other countries, the Russian Federation has adopted a sequential scheme of immunization against polio, IPV followed by OPV, since 2008 [32]. The first experimental series of Sabin-IPV were made by FSBSI “Chumakov FSC R&D IBP RAS” in 2015 [33]. The data of phase I and II clinical trials in adults of 18–60 years of age presented here showed low reagtogenicity and safety of the novel Sabin strain–based inactivated polio vaccine PoliovacSin comparable to the safety profile of Imovax Polio.

Immunogenicity study of vaccinees’ sera showed an increase in antibody titers in all participants, regardless of the vaccine used. To evaluate the spectrum of antibody response induced by PoliovacSin wild poliovirus strains were used in neutralization test as well as vaccine Sabin ones. The titers against strains Sabin 1, Sabin 2 and Mahoney did not differ significantly between PoliovacSin group and Imovax Polio group. However, the titers against strains Sabin 3, MEF, and Saukett tended to be a little lower in the group vaccinated with PoliovacSin than in the group vaccinated with Imovax Polio. Nonetheless, antibodies induced by PoliovacSin can neutralize not only Sabin viruses but the wild polioviruses as well. Therefore, the immunogenicity profile of the new vaccine can be considered comparable to Imovax Polio.

Overall, the results of the clinical trials in adults of 18–60 years of age allowed the recommendation of the PoliovacSin for clinical trials in children and infants for the purpose of subsequent registration of the vaccine in the Russian Federation for use within the framework of the National Calendar of Preventive Vaccinations.

## Figures and Tables

**Table 1:** Baseline characteristics of study participants who received PoliovacSin or Placebo. Phase I.

Characteristics	PoliovacSin Group	Placebo Group
Participants, no	30	30
Female/Male, no	16/14	11/19
Age, years, mean ± SD	37.4 ± 11.4 *	32.5 ± 11.4 *
Height, m, mean ± SD	1.72 ± 0.09 *	1.75 ± 0.11 *
Weight, kg, mean ± SD	74.6 ± 10.7 *	77.4 ± 9.5 *

* Differences between groups are statistically insignificant (*t*-test, *p* < 0.05).

**Table 2:** Adverse events caused by immunization with PoliovacSin or Placebo, overall and by severity grade.

Group	Participants, No	Adverse Events Registered from 0 to 7 Day After Vaccination											
		Local						Systemic					
		Grade 1		Grade 2		Grade 3		Grade 1		Grade 2		Grade 3	
		N	%	N	%	N	%	N	%	N	%	N	%
PoliovacSin	30	2	6.67	–	–	–	–	–	–	–	–	–	–
Placebo	30	– *	–	–	–	–	–	–	–	1	3.33	–	–
Total	60	2	3.33	–	–	–	–	–	–	1	1.67	–	–

* No reaction was recorded.

**Table 3:** Baseline characteristics of study participants who received PoliovacSin or Imovax Polio. Phase II.

Characteristics	PoliovacSin Group *	Imovax Group *
Participants, no	100	100
Female/Male, no	59/41	61/39
Age, years, mean ± SD	32.1 ± 9.3	31.7 ± 9.1
Female/Male 45 + years of age, no	8/8	6/4
Height, m, mean ± SD	1.71 ± 0.08	1.69 ± 0.08
Weight, kg, mean ± SD	73.4 ± 13.8	69.0 ± 12.1

* Differences between groups are statistically insignificant (*t*-test, *p* < 0.05).

**Table 4:** Adverse events caused by primary receipt of PoliovacSin or Imovax Polio, overall and by severity grade. Phase II.

Group	Participants, No	Adverse Events Registered from 0 to 7 Day After Vaccination											
		Local						Systemic					
		Grade 1		Grade 2		Grade 3		Grade 1		Grade 2		Grade 3	
		N	%	N	%	N	%	N	%	N	%	N	%
PoliovacSin	100	7	7.0	1	1.0	–	–	7	7.0	1	0.5	–	–
Imovax Polio	100	5	5.0	– *	–	–	–	5	5.0	–	–	–	–
Total	200	12	6.0	1	0.5	–	–	12	6.0	1	0.5	–	–

* No reaction was recorded.

## Data Availability

Raw data is available from the authors upon reasonable request.
